# Ovariotomy in the Eightieth Year

**Published:** 1901-03-23

**Authors:** 


### OVARIOTOMY IN THE EIGHTIETH YEAR.

The New York *Medical News* reports the removal, by Dr. Krusen, of cysts of both ovaries from a woman 80 years of age, the patient making a good recovery. The two cysts weighed together 15 pounds. A point worth noting was their slow growth, the patient having first noticed the abdominal tumour 26 years ago.

